# Cold Plasma Pretreatment Enhances Antioxidant Stability in Lime Fruit During Storage

**DOI:** 10.1002/fsn3.71091

**Published:** 2025-10-13

**Authors:** Ghazal Razazian, Mohsen Dalvi‐Isfahan, Saeideh Mohtashami

**Affiliations:** ^1^ Department of Food Science and Technology, Faculty of Agriculture Jahrom University Jahrom Fars Iran; ^2^ Department of Horticultural Science, Faculty of Agriculture Jahrom University Jahrom Fars Iran

**Keywords:** cold plasma, DBD, flavonoid, GLIDE, lime fruit, phenolic, postharvest treatment

## Abstract

Lime ( *Citrus aurantiifolia* ) is a nutrient‐rich citrus fruit with high antioxidant potential but a limited shelf life due to physiological and microbial deterioration. This study explores the efficacy of cold plasma (CP) pretreatment, including dielectric barrier discharge (DBD) and gliding arc (GLIDE) plasma, in enhancing the antioxidant properties and storage stability of lime fruit. The results demonstrate that CP treatment significantly influences key bioactive compounds, including vitamin C, total phenolics, flavonoids, and tannins, particularly in the peel. DBD plasma at 10 min exhibited the most pronounced enhancement of antioxidant compounds. Fourier‐transform infrared spectroscopy (FTIR) and scanning electron microscopy (SEM) analyses revealed surface chemical modifications and structural changes induced by CP, affecting enzymatic activity and bioactive compound release. Antioxidant activity exhibited a biphasic trend, with initial declines followed by a recovery phase, particularly in CP‐treated samples. These findings suggest that CP treatment, particularly DBD plasma, enhances the antioxidant profile of lime while extending its postharvest stability. The study highlights CP as a promising, nonchemical alternative for improving the nutritional properties of citrus fruits. Further optimization of plasma parameters could enhance commercial applications.

## Graphical abstract

This study investigates the use of cold plasma (CP) pretreatment, including dielectric barrier discharge (DBD) and gliding arc (GLIDE) plasma, to enhance the antioxidant properties and storage stability of lime. Results show that DBD plasma at 10 min significantly increases bioactive compounds, such as vitamin C and flavonoids, while extending the lime's shelf life, making CP a promising nonchemical method for improving citrus fruit quality.

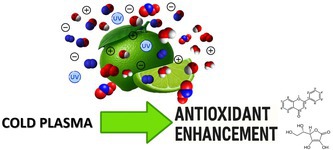

## 1 Introduction

Lime ( *Citrus aurantiifolia* ) is a widely consumed citrus fruit known for its rich nutritional profile and health‐promoting properties. Citrus is one of the most economically significant crops in Iran, with total citrus production estimated at approximately 5,613,130 tons during 2019–2020 (Bakhshandeh et al. 2022). Limes are an excellent source of vitamin C, flavonoids, and other bioactive compounds, which contribute to their strong antioxidant capacity. These properties make limes highly beneficial for human health, including immune system support, reduction of oxidative stress, and potential protective effects against chronic diseases (Huynh et al. 2023). However, despite their high nutritional value, limes have a relatively short shelf life due to their susceptibility to mechanical injury, physiological deterioration, water loss, and microbiological decay during harvest, transport, and storage (González‐Estrada et al. 2017).

To address these challenges, various preservation technologies have been employed to extend shelf life, such as modified atmosphere packaging (Navaratne and Sandaruwani 2014), edible coatings (Mohammadi et al. 2024), and chemical preservation (Li et al. 2023). While these methods can enhance freshness, they often have drawbacks such as potential health concerns, high costs, and environmental impact. Importantly, recent research has shown that proper postharvest treatments not only extend shelf life but can also enhance the nutritional value of fruits like lime, particularly by increasing their antioxidant content (Denoya et al. 2020). Consequently, there has been growing interest in innovative and sustainable postharvest treatments that both preserve fruit quality and boost nutritional value.

Among these emerging technologies, cold plasma (CP) has gained significant attention as a novel postharvest method capable of simultaneously extending shelf life and decontamination of fruits (Dalvi‐Isfahan and Mahmoodi‐Eshkaftaki 2024). Notably, CP treatment has been shown to induce stress responses in plant tissues, stimulating the biosynthesis of defensive secondary metabolites with antimicrobial and antioxidant properties. While CP technology has been extensively studied for its role in food decontamination (Zhou et al. 2024), relatively recent investigations have examined its efficacy in enhancing nutritional quality, increasing antioxidant compound levels, or assessing its impact on the physicochemical properties of fruits and vegetables (Zargarchi et al. 2024). For instance, in kumquat (*Citrus japonica*), intermittent corona discharge plasma jet treatment (8 kV for 90 s) significantly increased vitamin C and total phenolic content while maintaining stable antioxidant activity during storage (Puligundla et al. 2018). Similarly, CP treatment (900 W for 10 min) of mandarin ( *Citrus reticulata* ) enhanced the total phenolic content in the peel, thereby improving its antioxidant capacity (Won et al. 2017). CP treatment has also demonstrated notable effects on non‐citrus fruits. In blueberries (*Vaccinium* sp.), application of high‐voltage dielectric barrier discharge (DBD) plasma (80 kV for 1–5 min) significantly increased total phenolic and flavonoid contents, as well as vitamin C levels (Sarangapani et al. 2017). In pomegranate juice ( *Punica granatum* ), exposure to a constant jet plasma stream resulted in elevated anthocyanin content, correlating with enhanced antioxidant activity (Bursać Kovačević et al. 2016).

The effectiveness of CP treatment in enhancing the antioxidant profile of fruits could be attributed to multiple mechanisms, including increased extractability of phenolic compounds, activation of the enzyme responsible for flavonoid biosynthesis (phenylalanine ammonia‐lyase), and reduction of oxidative degradation by reactive oxygen species. Additionally, CP can induce structural modifications in plant tissues that facilitate the release of bound phenolic compounds, thereby enhancing their bioaccessibility (Dalvi‐Isfahan and Mahmoodi‐Eshkaftaki 2024; Zargarchi et al. 2024). Among the different CP technologies, DBD plasma and gliding arc (GLIDE) plasma stand out for their potential applications in food preservation. DBD plasma, known for its uniform discharge and moderate energy input, is widely applied in food processing for microbial inactivation and surface biochemical modifications. Operating under atmospheric pressure, DBD plasma generates reactive oxygen and nitrogen species (RONS) that can influence enzymatic activities and oxidative stability, potentially impacting the storage quality of lime. In contrast, GLIDE operates at higher energy levels and produces a greater density of reactive species, leading to stronger oxidative effects. This may result in enhanced microbial inactivation but also cause significant biochemical alterations, depending on treatment conditions (Birania et al. 2022; Yawut et al. 2024). This study aims to investigate the novel application of two distinct CP sources—DBD (low intensity and long exposure) and GLIDE arc (high intensity and short exposure)—and their comparative effectiveness in enhancing the antioxidant stability of lime fruit during storage. To the best of our knowledge, this is the first research to directly compare these two plasma sources in terms of their impact on the antioxidant properties of lime fruit. Additionally, the study examines the effect of storage duration on antioxidant stability. By exploring the combined effects of CP pretreatment and storage conditions, this research aims to provide unique insights into the most effective strategy for preserving the antioxidant properties of lime fruit, thereby improving its shelf life and commercial value.

## 2 Materials and Methods

### 2.1 Materials

Mexican lime fruits ( *Citrus aurantiifolia* ), locally known as Omani lime, were harvested from a single non‐grafted tree in Jahrom, Fars Province, Iran, to minimize varietal variation. The fruits were transported to the laboratory within approximately 1 h and 30 min at ambient temperature (20°C), washed, and sorted to remove any damaged ones. To maintain freshness, they were placed in micro‐perforated zip‐sealed bags and stored in an incubator at 7°C–8°C throughout the experiment to prevent chilling injury.

### 2.2 DBD‐CP Pretreatment

In this study, a DBD CP system (Enhancedtech 15I, Kavoshyaran Co. LTD., Iran) was employed using ambient air as the carrier gas. The technical specifications of the DBD setup were previously described in detail by Ghomi et al. (2017). The system utilized a dual power supply configuration: the first power source operated at 0–25 kV and 50 Hz, while the second provided 0–10 kV at 6 kHz. The lower electrode, constructed from stainless steel, was affixed to a glass bowl to ensure uniform plasma distribution across varying discharge gaps. During operation, the top electrode was energized using the high‐frequency (6 kHz, 10 kV) power source. The lower electrode, on the other hand, was connected to the low‐frequency (50 Hz, up to 25 kV) supply. The system incorporated two parallel electrodes, each measuring 22 cm by 15 cm.

**FIGURE 1:**
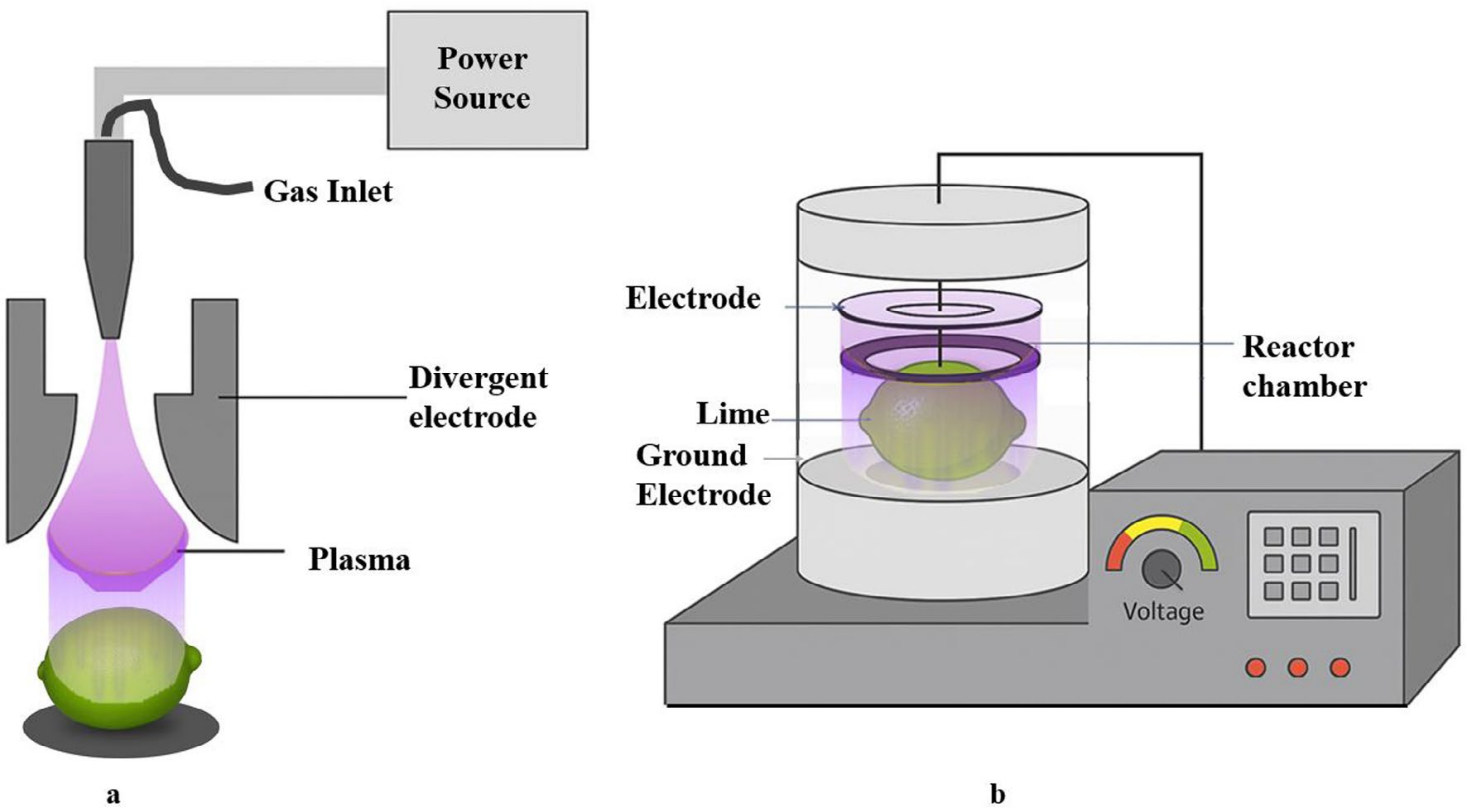
Schematic representation of the cold plasma pretreatment setup: (a) Glide and (b) DBD.

To optimize treatment conditions and prevent visible surface damage to the lime, preliminary trials were conducted to determine suitable exposure durations. Based on these trials and previous studies (Ahmadnia et al. 2021; Tappi et al. 2016), three treatment times were selected: 4 min (2 min per side), 6 min (3 min per side), and 10 min (5 min per side). After treatment, the fruits were stored under controlled conditions (in a cold incubator and 85% relative humidity), and quality assessments were conducted at 10‐day intervals over a 30‐day period. For clarity in data representation, the samples were designated as DBD‐4 min, DBD‐6 min, and DBD‐10 min, corresponding to their respective treatment durations. The CP pretreatment setup is schematically represented in Figure 1.

### 2.3 Glide Arc CP Pretreatment

A glide arc discharge CP system was employed, operating at 400 watts with a current of 2 A and an electrode gap of 2 cm. Lime fruits were exposed to plasma for 90 s (45 s per side), 120 s (60 s per side), and 150 s (75 s per side). The distance between the tip of the electrode and the fruit surface was consistently maintained at 30 mm (Figure 1). Untreated fruits were used as the control group. The selection of exposure durations was informed by previous studies and preliminary trials to ensure effective treatment without causing visible surface damage (Akaber et al. 2024). Following glide arc plasma treatment, the fruits were stored under the same conditions as those treated with the DBD system, and quality assessments were performed at 10‐day intervals over a 30‐day storage period. For clarity in data analysis and presentation, samples were labeled GLIDE‐90 s, GLIDE‐120 s, and GLIDE‐150 s, corresponding to their respective treatment durations.

### 2.4 Fourier Transform Infrared (FTIR) Spectroscopy

In this study, the FTIR spectroscopy method described by Misra et al. (2015) was employed. The effects of CP pretreatments, specifically DBD and GLIDE, on the samples were analyzed using an FTIR spectrometer (Tensor II, Bruker, Germany) equipped with an attenuated total reflectance (ATR) accessory. Spectral data were recorded across a wavenumber range of 400–4000 cm^−1^ with a resolution of 4 cm^−1^.

### 2.5 Scanning Electron Microscopy (SEM)

The lime peel samples, including the control, DBD‐10 min, and GLIDE‐150 s treatments, were analyzed using SEM to examine surface morphological changes. Prior to imaging, the samples were coated with a thin layer of gold using a Quorum Technologies Q150R‐ES gold sputter coater (UK) to enhance conductivity. SEM images were captured at 500× magnification using a TESCAN Vega3 scanning electron microscope (TESCAN, Czech Republic).

### 2.6 Vitamin C Content

Ascorbic acid content in lime fruit was determined according to the method described by Najwa and Azlan (2017). Briefly, the fruit was homogenized with 6% metaphosphoric acid. A 10 mL portion of the homogenate was diluted to 100 mL with 3% metaphosphoric acid and filtered under vacuum. A 10 mL aliquot of the filtrate was then titrated with 2,6‐dichlorophenol‐indophenol dye to a faint pink endpoint. The vitamin C concentration was calculated based on the volume of dye consumed and a standard calibration curve.

### 2.7 Determination of Antioxidant Compounds

#### 2.7.1 Extraction of Active Compounds

Lime juice and peel extracts were prepared for biochemical analysis. Lime juice was centrifuged at 4600 rpm for 10 min, and the clear supernatant was collected for further analysis.

For peel extraction, the peel was first dried at room temperature and finely ground. Extraction was performed using 70% methanol at a 5:1 (v/w) solvent‐to‐sample ratio. Specifically, 5 g of peel powder was mixed with 25 mL of 70% methanol in a Falcon tube, then agitated on a shaker at 250 rpm for 22 h. After centrifugation at 4600 rpm for 10 min, the clear supernatant was collected as the peel extract. All extracts were stored at −20°C for subsequent experiments.

#### 2.7.2 Determination of Antioxidant Activity (DPPH Assay)

Antioxidant activity was assessed using the 2,2‐diphenyl‐1‐picrylhydrazyl (DPPH) assay, following the method of Oke et al. (2009), with minor modifications. A 0.004% DPPH solution (prepared by dissolving 0.004 g DPPH in 100 mL methanol) was mixed with 100 μL of lime juice or peel extract. The mixture was vortexed for 5 s, incubated in the dark at room temperature for 30 min, and the absorbance was measured at 517 nm using a spectrophotometer. A blank control was prepared by replacing the extract with methanol. Antioxidant activity (%) was calculated using the following formula:

$$\mathrm{AOA}\%=\left(\left(A_{0}-A_{1}\right)\right)/A_{0}\times 100$$

where *A*_0_ is the absorbance of the control and A_1_ is the absorbance of the sample.

#### 2.7.3 Determination of Total Phenolic Content (TPC)

Total phenolic content was determined using the Folin–Ciocalteu method (Wojdyło et al. 2007). 100 μL of extract was mixed with 200 μL of 50% Folin–Ciocalteu reagent, followed by the addition of 2 mL distilled water. After 3 min, 1 mL of 20% sodium carbonate was added. The mixture was incubated in the dark at room temperature for 30 min, and absorbance was recorded at 765 nm. A standard curve was prepared using gallic acid (0–400 ppm), and results were expressed as mg gallic acid equivalent per gram of dry weight (mg GAE/g DW).

#### 2.7.4 Determination of Total Flavonoid Content (TFC)

Total flavonoid content was measured following the method of Menichini et al. (2009). 500 μL of extract was mixed with 150 μL of 5% sodium nitrite. After 5 min, 300 μL of 10% aluminum chloride was added. After another 6 min, 2 mL of 0.5 M sodium hydroxide was added, and the final volume was adjusted with 250 μL distilled water. Absorbance was measured at 510 nm, and a standard curve was prepared using quercetin (0–400 ppm). Results were expressed as mg quercetin equivalent per gram of dry weight (mg QE/g DW).

#### 2.7.5 Determination of Flavones and Flavonols

Flavone and flavonol content was measured following the method of Popova et al. (2004), with slight modifications. 200 μL of extract was mixed with 2 mL methanol and 100 μL of 5% aluminum chloride, then diluted to 5 mL with distilled water. After incubation at room temperature for 30 min, absorbance was measured at 425 nm. A quercetin standard curve was used to quantify flavone and flavonol content.

#### 2.7.6 Determination of Tannin Content

Tannin content was determined using the vanillin‐HCl method (Broadhurst and Jones 1978). 250 μL of extract was mixed with 1.5 mL of 4% vanillin reagent (prepared in methanol) and 750 μL of concentrated HCl. The solution was mixed thoroughly and incubated in the dark at room temperature for 15 min. Absorbance was measured at 500 nm, and tannin content was expressed as catechin equivalents (CE) per 100 g of sample.

### 2.8 Determination of Total Carbohydrate Content

Total carbohydrate content was measured following the anthrone method (Yemm and Willis 1954). 100 μL of extract (referring to either the lime juice or lime peel extract) was mixed with 3 mL of 0.2% anthrone reagent, heated in a boiling water bath for 10 min, then cooled in ice water. Absorbance was measured at 630 nm, and a glucose standard curve (0–400 ppm) was used to quantify total carbohydrates, expressed as mg glucose per gram of dry weight (mg/g DW).

### 2.9 Statistical Analysis

In this study, a split‐plot design within a completely randomized design (CRD) was employed to investigate the effects of CP pretreatment and storage duration, as well as their interaction. The CP pretreatment was applied at three exposure times for each plasma type: DBD for 4, 6, and 10 min, and GLIDE discharge for 90, 120, and 150 s. Analysis of variance (ANOVA) was performed using the General Linear Model in SPSS (version 26), with statistical significance set at *p* ≤ 0.05. Tukey's test was used for mean comparison among pretreatments. The results are presented as mean ± standard deviation (SD), based on three replicates. Graphs were generated using Microsoft Excel.

## 3 Results and Discussion

### 3.1 FTIR Analysis

**FIGURE 2:**
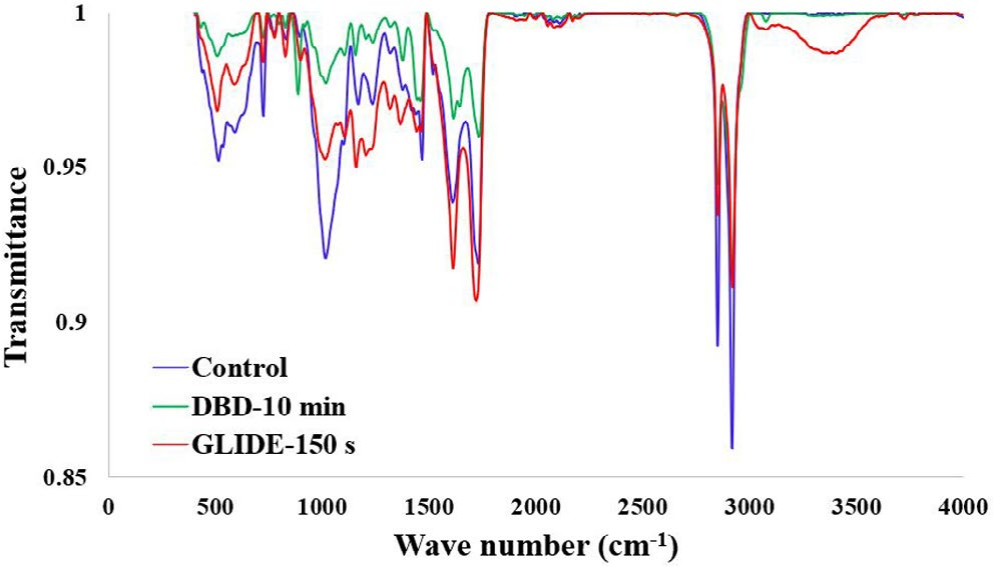
FTIR spectra of lime fruit over 400–4000 cm^−1^ region.

FTIR spectroscopy was employed to investigate the chemical modifications on the lime fruit surface following CP treatments. Figure 2 shows the FTIR spectra for the control, DBD‐10 min, and GLIDE‐150 s samples. The spectra predominantly displayed bands characteristic of plant cuticles and polysaccharides. A broad band around 3300 cm^−1^ corresponds to O–H stretching vibrations from hydroxyl groups in cellulose, pectin, and adsorbed water (Abidi et al. 2014), while peaks at 2918 cm^−1^ and 2850 cm^−1^ are due to the stretching vibrations of methylene groups (–CH_2_) in epicuticular waxes and cutin (Alashti et al. 2024). The C=O stretching band at approximately 1735 cm^−1^ is associated with ester groups in pectin and cutin, and bands around 1240 cm^−1^ and 1030 cm^−1^ correspond to C–O stretching and C–O–C glycosidic bonds in polysaccharides (Kurniawan et al. 2020). A noticeable change between the control and plasma‐treated samples occurs in the 2800–3000 cm^−1^ region, where both DBD and GLIDE treatments caused a reduction in the intensity of the aliphatic C–H stretching peaks at 2918 cm^−1^ and 2850 cm^−1^. This suggests partial removal or degradation of the hydrophobic wax layer on the lime peel surface.

However, minimal changes were observed in other spectral regions. The C=O ester peak (~1735 cm^−1^) and polysaccharide C–O bands (~1240 and 1030 cm^−1^) remained largely unchanged after plasma exposure, indicating that the plasma treatments did not cause significant oxidation or alter the underlying polysaccharide structure. This confirms that the plasma effects are confined to the surface, with the deeper chemical composition largely preserved, in line with findings from Misra et al. (2015) on plasma‐treated strawberries. Additionally, no significant changes are observed in the C=O stretching (~1727 cm^−1^) and C–O stretching (1000–1250 cm^−1^) regions, implying minimal oxidation or incorporation of oxygen species. This contrasts with the findings of Grzegorzewski et al. (2010) on RF‐glow discharge plasma‐treated lamb's lettuce, where significant oxidation and the formation of new oxygen‐containing functional groups were reported. The lack of substantial oxidation in this study can be attributed to differences in plasma type, gas composition, and the thicker cuticle of lime fruit, which likely acts as a barrier to oxygen incorporation. Overall, FTIR analysis reveals that both GLIDE and DBD treatments primarily affect the aliphatic, waxy components of the lime cuticle, with minimal impact on the underlying polysaccharide structure.

### 3.2 SEM Analysis

**FIGURE 3:**
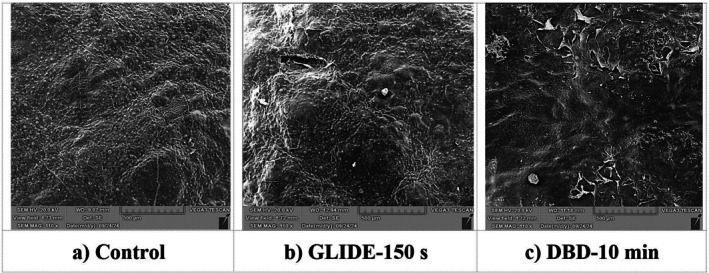
SEM images of lime peel samples under different treatments: (a) Control, (b) GLIDE plasma‐treated for 150 s, and (c) DBD plasma‐treated for 10 min.

Figure 3 presents SEM images of lime peel samples subjected to different plasma treatments. The control sample exhibits a smooth surface with fine natural roughness, characteristic of the natural ultrastructure, with a continuous epicuticular wax layer and evenly distributed stomata (15–25 μm in diameter). The structure was well preserved, showing only minimal micro‐fissures.

The GLIDE plasma‐treated sample shows increased surface roughness, with noticeable cracks and small pores, suggesting a mild etching effect. While the underlying topography remains largely intact, the waxy layer is partially eroded, and larger cracks (100–150 μm in length) are observed, indicating more intense plasma interaction in localized areas. Despite these changes, the peel's structural integrity is largely preserved, with the primary effect on the waxy layer, exposing underlying components. In contrast, the DBD plasma‐treated sample demonstrates the most pronounced surface modifications, characterized by extensive cracking and fragmentation of the outer layer. The surface exhibits large flakes (150–200 μm in size) of the epicuticular wax and cuticle delaminating, pointing to a more aggressive etching and ablative effect, which results in the breakdown of hydrophobic compounds such as cutin and wax. Overall, the SEM analysis shows that both plasma treatments alter the lime peel surface, but with different intensities. GLIDE plasma induces moderate, uniform surface etching, while DBD plasma leads to extensive and destructive modifications. These differences in surface morphology are expected to affect properties such as hydrophilicity, gas permeability, and microbial attachment, emphasizing the importance of optimizing plasma treatment conditions to control the degree of surface modification for specific postharvest outcomes. These findings are consistent with those of Akaber et al. (2024) and Huang et al. (2019), who reported that plasma treatment enhanced both the quantity and size of cavities or cracks, with prolonged exposure times resulting in more significant changes.

### 3.3 Vitamin C Content of Lime Juice

**FIGURE 4:**
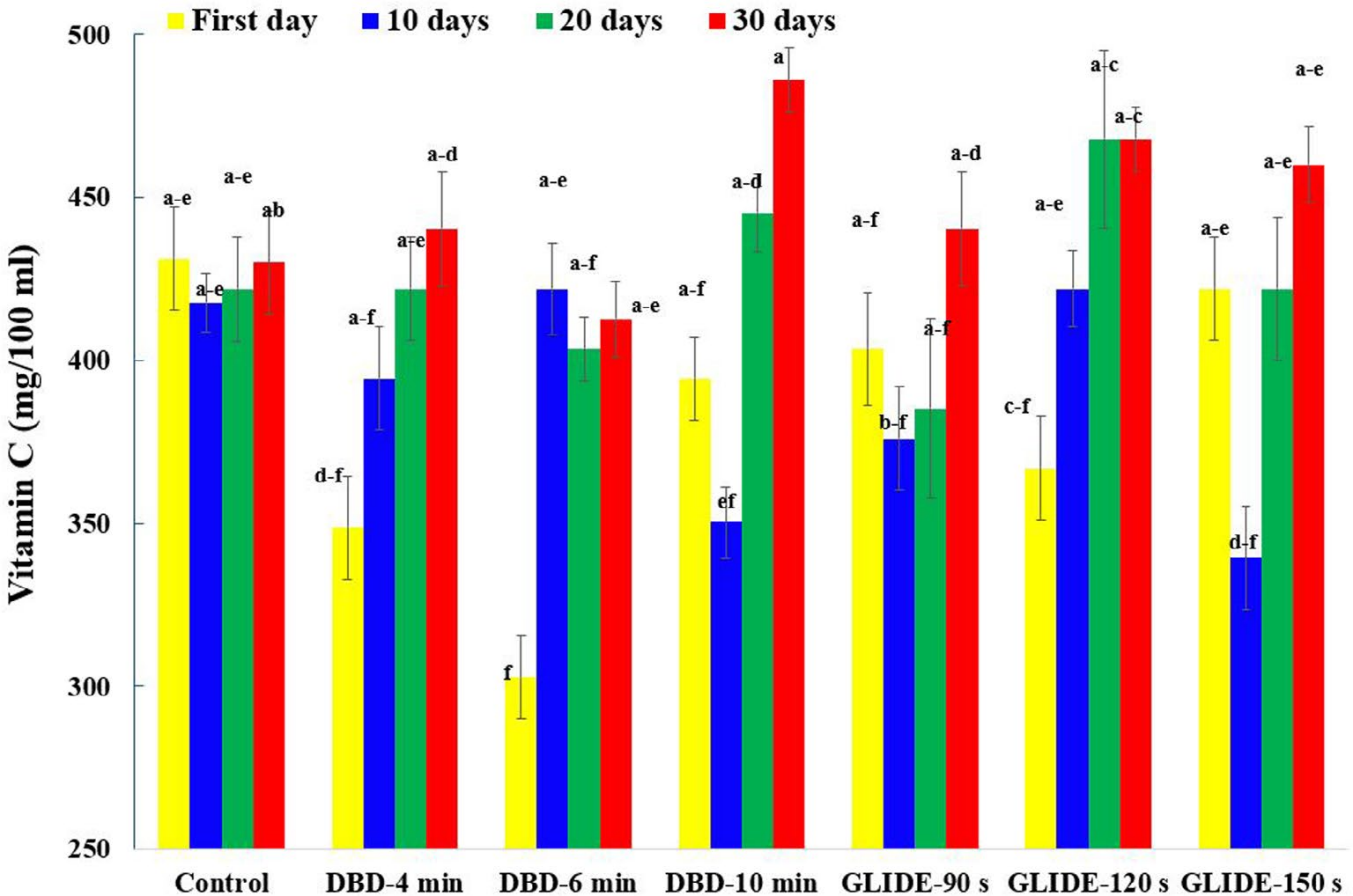
Ascorbic acid content of lime fruit juice in control and plasma‐treated samples during storage period.

Ascorbic acid, a key antioxidant in fresh fruits, was evaluated under different plasma treatment conditions and storage durations (Figure 4). Statistical analysis showed that both storage time and plasma treatment intensity significantly affected ascorbic acid levels (*p* < 0.05). After 30 days, samples treated with DBD for 10 min and GLIDE for 150 s showed increases in vitamin C content of over 13% and 7%, respectively, compared to the control. These findings align with Sarangapani et al. (2017), who reported similar results in DBD‐treated blueberries. Overall, the changes in vitamin C content suggest a nonlinear relationship between plasma treatment parameters and ascorbic acid retention, influenced by both protective and degradative mechanisms. The generation of nitric oxide (NO) during plasma treatment plays a key role, which regulates the ascorbate‐glutathione cycle and enhances the conversion of dehydroascorbic acid to ascorbic acid. When the rate of regeneration exceeds degradation, vitamin C content increases; however, degradation surpasses regeneration, as noted by Dong and Yang (2019). Figure 4 also shows a transient decrease in ascorbic acid immediately after plasma treatment, likely due to the generation of reactive oxygen and nitrogen species (RONS). In subsequent weeks, both plasma‐treated and control samples exhibited a rise in vitamin C content, suggesting that prolonged cold storage may enhance vitamin C levels through natural metabolic and enzymatic processes. Plasma‐treated samples, particularly those exposed to DBD, showed a more pronounced increase, possibly as a response to oxidative stress.

### 3.4 Total Phenolic Content in Lime Juice and in Lime Peel

**FIGURE 5:**
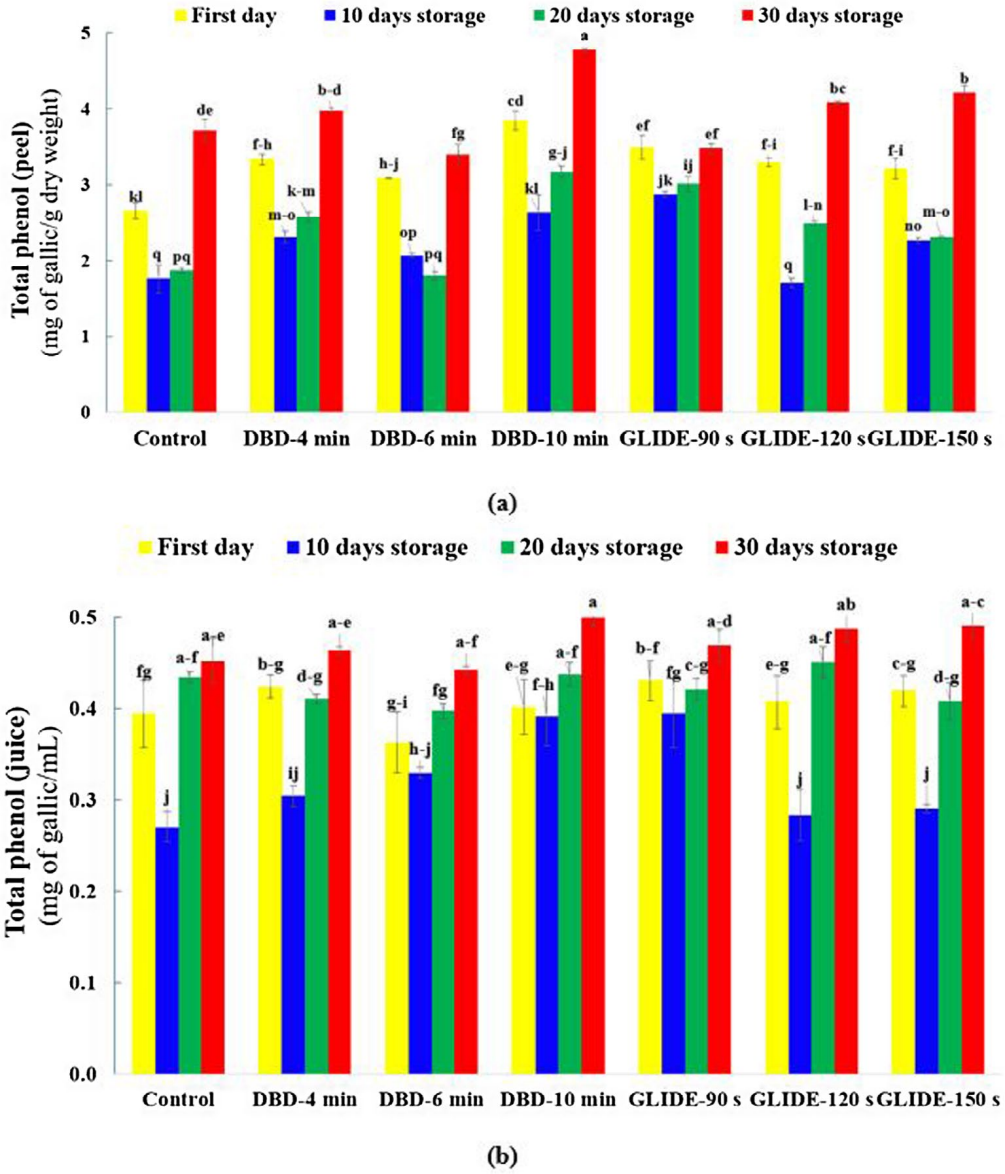
Total phenolic content, expressed as gallic acid equivalents, in control and plasma‐treated samples during the storage period: (a) Lime fruit peel and (b) lime fruit juice.

The total phenolic content (TPC) in lime juice and peel during various storage periods and under CP pretreatments (DBD and GLIDE) is presented in Figure 5a,b, respectively. On the first day, TPC levels across all treatments were relatively low and not significantly different (*p* > 0.05). However, as storage progressed, a significant increase in TPC was observed, particularly in CP‐treated samples of lime peel. This rise may be attributed to stress‐induced physiological responses or the activation of phenolic biosynthesis pathways. These results may also reflect the activation of the phenylpropanoid pathway by CP, which promotes the biosynthesis of phenolic compounds through enzymes such as Phenylalanine Ammonia Lyase (PAL) and Cinnamate‐4‐Hydroxylase (C4H), leading to an increased accumulation of phenolics (Fernandes and Rodrigues 2021).

Among the treatments, DBD (especially at 10 min) and GLIDE (at 150 s) showed the greatest TPC enhancement, suggesting that both plasma types effectively boost TPC, though the extent depends on exposure duration. Similarly, Dasan and Boyaci (2018) found that CP treatment of orange juice for 120 s significantly increased TPC compared to the control.

TPC in lime peel was higher than in juice, which is expected as the peel serves as the primary defense barrier against environmental stressors. Being a surface treatment, CP has a more pronounced effect on the peel than the juice. Won et al. (2017) reported that plasma‐generated UV radiation can penetrate the epidermal cells of citrus fruits like mandarins, stimulating phenolic compound biosynthesis and enhancing antioxidant activity in the peel. However, due to the peel's thickness, plasma penetration into the inner fruit is limited, leading to minimal changes in juice TPC. As shown in Figure 5a, peel TPC generally increased over time, particularly by Day 30. This trend may result from plasma‐generated ROS and UV radiation promoting phenolic accumulation. CP treatment induces ROS generation, which acts as a signaling molecule and triggers the fruit's antioxidant systems, including the upregulation of antioxidant enzymes that mitigate oxidative stress (He et al. 2025; Li, Li, Ji, et al. 2019; Li, Li, Han, et al. 2019). The highest TPC was recorded in the DBD treatment at 10 min after 30 days, with a 25% increase compared to the first day. Although GLIDE also led to TPC enhancement over time, the increase was less pronounced. The highest TPC in GLIDE‐treated samples was seen at 150 s. Overall, longer plasma exposure in both DBD and GLIDE treatments resulted in greater ROS generation, which likely triggered defense responses and phenolic synthesis. Sreelakshmi et al. (2024) reported increased TPC in apples following CP treatment, linked to upregulation of key enzymes in the shikimate, pentose phosphate, and phenylpropanoid pathways. In summary, limes treated with CP and stored at low temperatures exhibited a gradual increase in TPC over time. This suggests that the combined effect of cold storage and plasma treatment contributes to the stabilization and enhancement of phenolic compounds.

### 3.5 Total Flavonoid Content in Lime Juice and Peel

**FIGURE 6:**
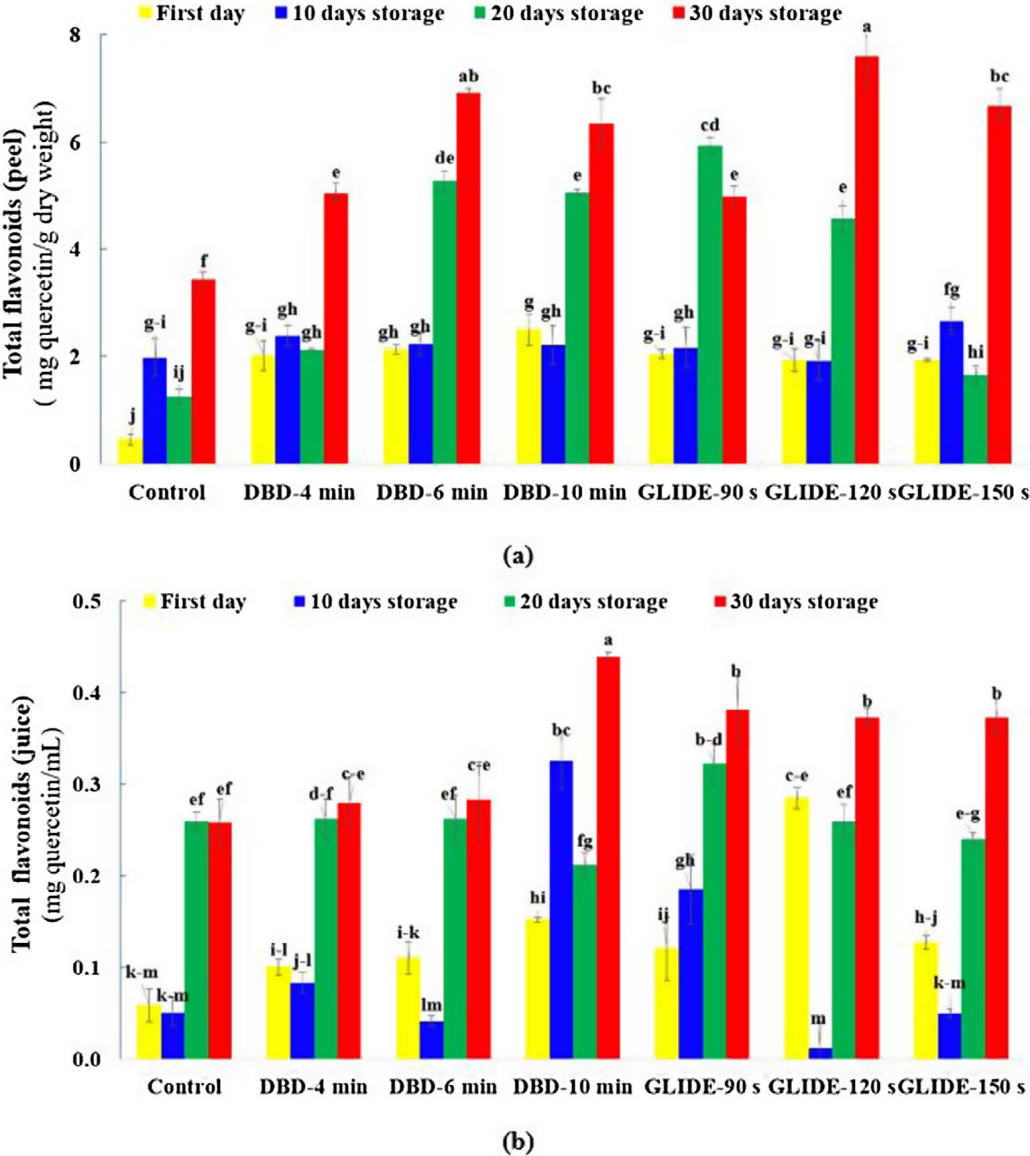
Total flavonoid content, expressed as quercetin equivalents, in control and plasma‐treated samples during the storage period: (a) Lime fruit peel and (b) lime fruit juice.

Figure 6a,b illustrate the changes in total flavonoid content in lime peel and juice during storage under the influence of two CP pretreatments (DBD and GLIDE). According to ANOVA, the effects of storage duration, plasma treatment, and their interaction were statistically significant (*p* < 0.05). A gradual increase in flavonoid content was particularly evident in plasma‐treated samples over time. The highest flavonoid levels were recorded after 30 days of storage in the plasma‐treated groups. This increase is likely due to elevated ROS generated by CP, which can stimulate flavonoid biosynthesis and cause structural modifications in these compounds. In contrast, control samples consistently exhibited the lowest flavonoid content throughout storage. The ROS produced during plasma treatment also act as signaling molecules, activating stress‐responsive pathways in the fruit, which further enhance flavonoid biosynthesis and contribute to increased antioxidant capacity (He et al. 2025; Li, Li, Ji, et al. 2019; Li, Li, Han, et al. 2019).

Among the treatments, DBD plasma at 10 min of exposure significantly enhanced flavonoid levels, especially by Day 30, suggesting that longer exposure times promote greater accumulation. Similarly, GLIDE treatment at 90, 120, and 150 s led to a substantial increase in flavonoid content, in some cases surpassing that of DBD‐treated samples. The 120‐s GLIDE treatment produced the highest flavonoid concentration, showing an approximate 120% increase in flavonoid content compared to the control after 30 days.

These findings align with previous research by Li, Li, Han, et al. (2019), who reported that plasma treatment enhances the biosynthesis of metabolites involved in the phenylpropanoid and flavonoid pathways in fresh strawberries. The enhancement in flavonoid content may be attributed to several factors, including degradation of cell wall polysaccharides, the indirect effects of plasma‐generated UV radiation and shock waves, and activation of phenylalanine ammonia‐lyase, an enzyme associated with the release of bound phenolics (Sarangapani et al. 2017).

### 3.6 Flavones and Flavonols in Lime Peel and Juice

**FIGURE 7:**
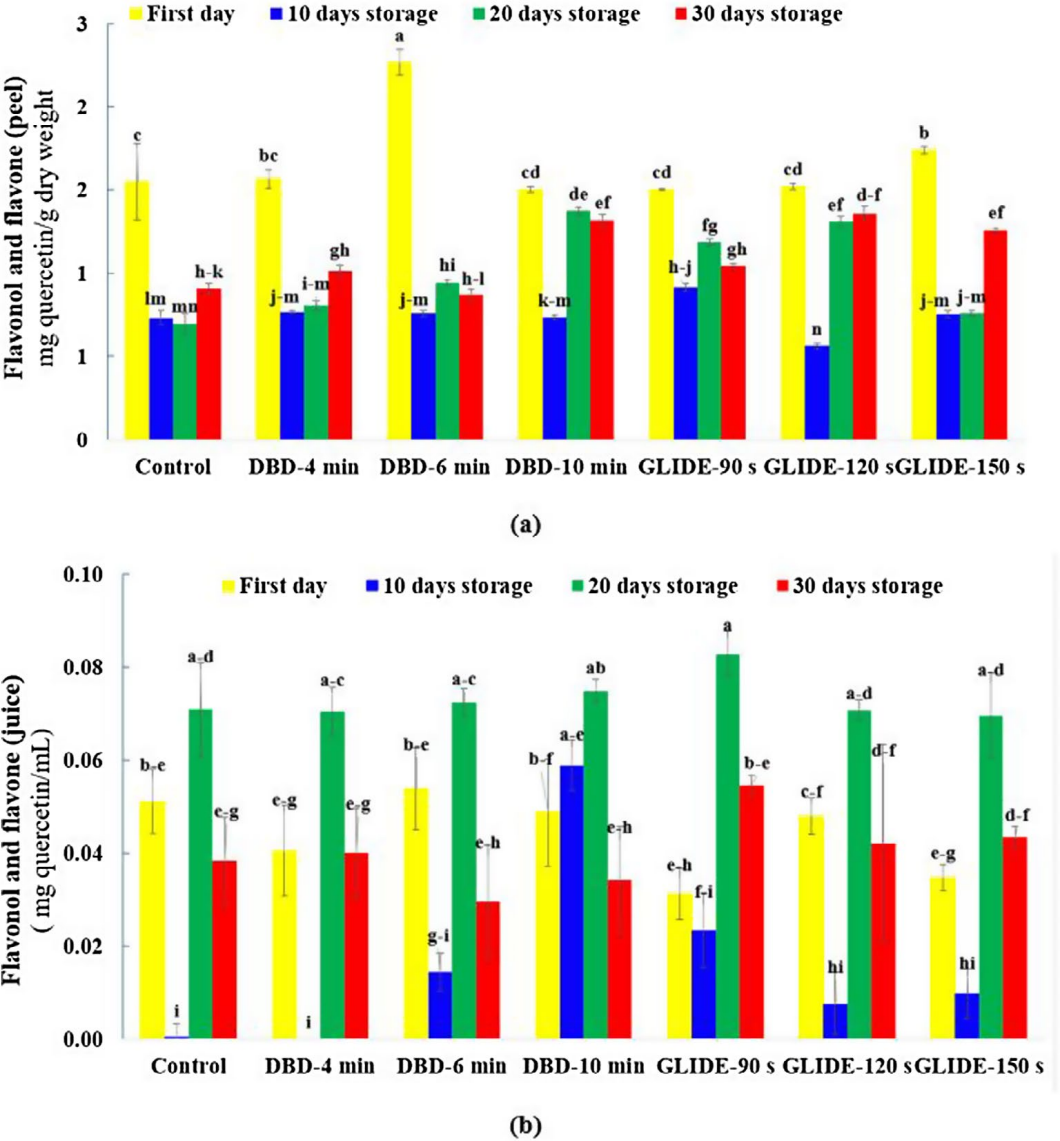
Total flavonol and flavone content, expressed as quercetin equivalents, in control and plasma‐treated samples during the storage period: (a) Lime fruit peel and (b) lime fruit juice.

As shown in Figure 7a,b, the highest flavone and flavonol content in lime peel was observed on the first day following plasma treatment. This was followed by a decreasing trend over time, with a slight increase toward the end of the storage period. The final increase may be related to ripening‐associated changes in peel color, driven by chlorophyll degradation and the accumulation of yellow pigments such as carotenoids and flavonoids.

Plasma treatment enhances the antioxidant capacity of plant tissues by generating ROS and RNS, which can break covalent bonds and trigger chemical reactions such as oxidation, reduction, hydrolysis, and dehydration. The dominant reaction depends on plasma type, power intensity, gas composition, and treatment duration. In this study, DBD plasma was applied for longer durations (up to 10 min), potentially allowing for gradual oxidative modifications and dehydration, while GLIDE plasma, applied for shorter durations (up to 150 s), generated more intense localized reactive species, which could favor hydrolysis‐driven modifications. Under these conditions, the late‐stage increase in flavonol content may be attributed to plasma‐induced stress responses, which activate enzymatic pathways involved in flavonoid biosynthesis (Fernandes and Rodrigues 2021).

Increased ROS production during CP treatment likely contributes to the activation of antioxidant enzymes such as superoxide dismutase (SOD) and catalase (CAT), which further improve the antioxidant capacity of the peel (Zargarchi et al. 2024).

On the first day, the highest flavone and flavonol content in the peel was recorded in the DBD‐treated sample at 6 min. Despite the overall decline during storage, the DBD‐10 min treatment retained the highest flavonoid levels at 20 and 30 days compared to the control. In the GLIDE‐treated samples, the highest initial content was observed at 150 s, and although levels declined over time, they remained higher than the control by Day 30.

A similar trend was observed in lime juice. The highest flavonoid content appeared after 20 days, but the decline was more rapid than in the peel—likely due to greater susceptibility to oxidation and enzymatic degradation in the juice matrix. Notably, the GLIDE‐150 s and DBD‐10 min treatments showed better flavonoid retention than the control by the end of the storage period, suggesting that plasma‐induced biochemical modifications may help stabilize flavonoids.

### 3.7 Antioxidant Activity in Lime Peel and Juice

**FIGURE 8:**
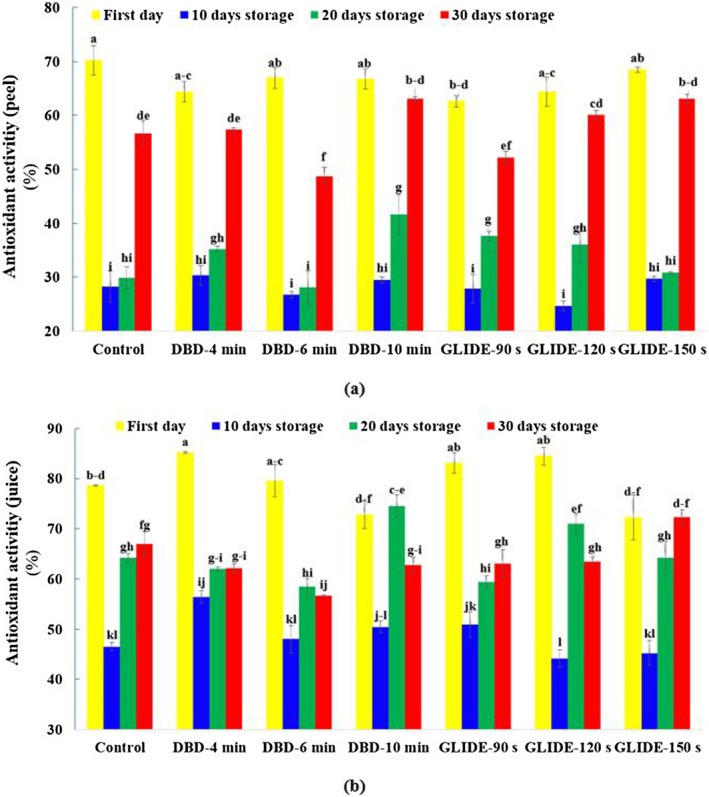
Antioxidant activity evaluated by the DPPH method in control and plasma‐treated samples during the storage period: (a) Lime fruit peel and (b) lime fruit juice.

Figure 8a,b shows the changes in nonenzymatic antioxidant activity in lime during storage under the influence of two CP pretreatments (DBD and GLIDE). ANOVA results indicated that plasma treatment type, storage duration, and their interaction significantly affected antioxidant levels (*p* < 0.05). On the first day of storage, antioxidant activity was highest across all treatment groups. As storage progressed, a gradual decline was observed, likely due to oxidation and enzymatic degradation by polyphenol oxidase and peroxidase (Lin et al. 2020). The most notable decrease occurred by Day 10 in the peel. However, from Day 20 onward, antioxidant levels began to recover in most plasma‐treated samples. By Day 30, these samples exhibited significantly higher antioxidant activity than the control, with the highest levels found in DBD‐10 min and GLIDE‐150 s treatments—approximately 12% higher than the untreated control. The biphasic trend in the control sample aligns with Rapisarda et al. (2008), where cold storage (6°C ± 1°C) affected vitamin C, phenolics, and antioxidant activity in orange genotypes. These results also support previous studies, such as Li, Li, Ji, et al. (2019), who observed increased antioxidant activity in CP‐treated fresh‐cut pitaya, attributing this enhancement to phenolic biosynthesis stimulation via the phenylpropanoid pathway, ROS signaling activation, ATP production increase, and microbial growth inhibition—all of which help preserve antioxidant compounds. Similarly, Chen et al. (2020) reported that CP treatment enhances and stabilizes antioxidant levels in fruits and vegetables. In conclusion, CP pretreatment appears to be a promising method for improving antioxidant stability in lime during storage, contributing to extended shelf life and better nutritional quality.

### 3.8 Total Carbohydrate Content in Lime Peel and Juice

**FIGURE 9:**
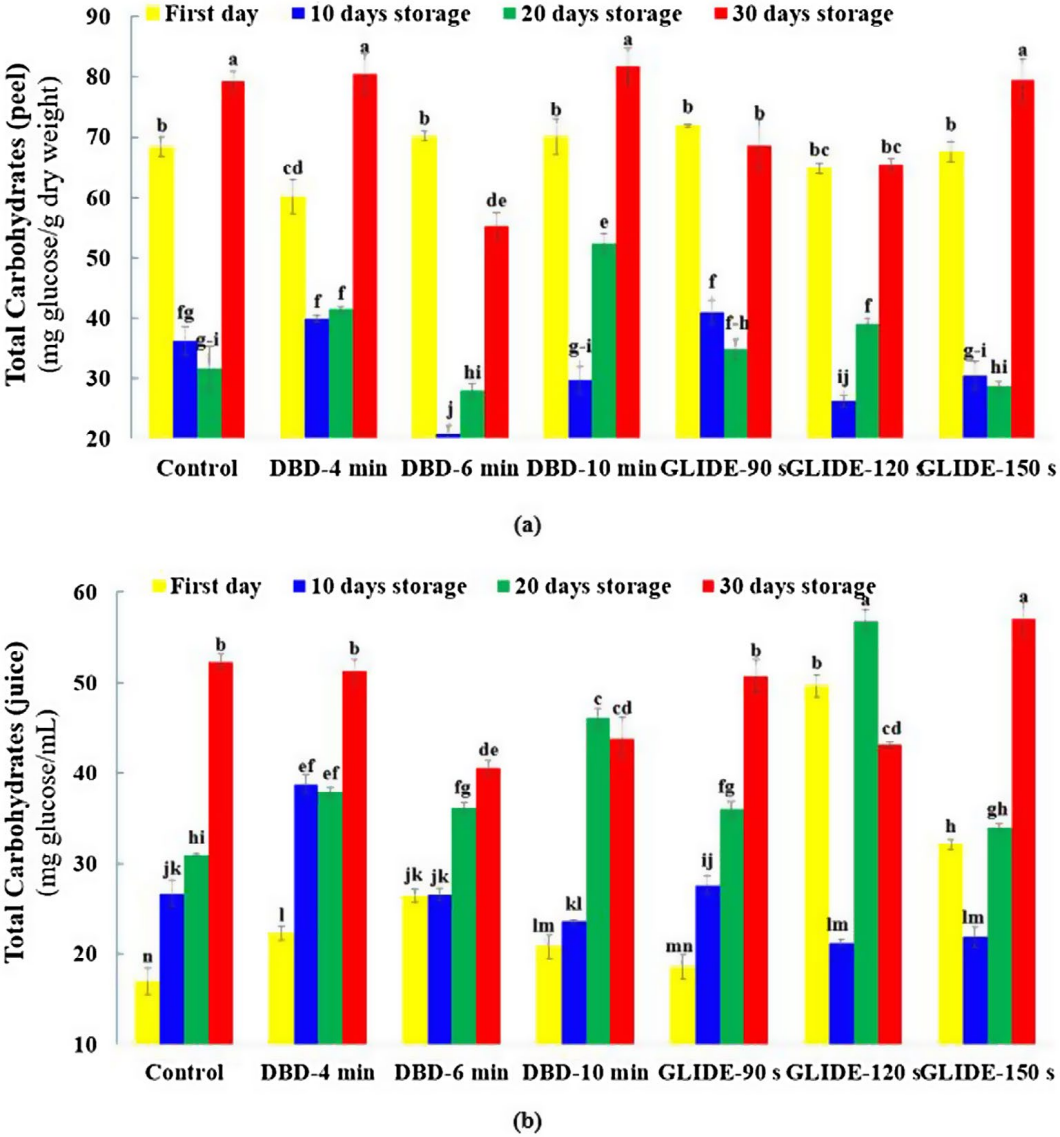
Total carbohydrate content, expressed as glucose equivalents, in control and plasma‐treated samples during the storage period: (a) Lime fruit peel and (b) lime fruit juice.

The total carbohydrate content in lime peel and juice showed distinct trends during cold storage following DBD and GLIDE plasma treatments (Figure 9). In the peel, a biphasic trend was observed. There was a sharp decline in carbohydrate levels during the first 10 days, followed by a gradual increase after 20 days. This suggests an initial carbohydrate depletion due to respiration and metabolic activity, with later accumulation potentially linked to sugar mobilization and cell wall modifications. In contrast, the juice showed a continuous increase in carbohydrate content from the first day of storage, unlike the peel. Among all treatments, the control samples showed a consistent carbohydrate decline in the peel. DBD‐treated samples exhibited an initial decline in the peel, with a stronger recovery at longer exposure times. In contrast, in the juice, carbohydrate content increased at shorter exposure durations but fluctuated over time. GLIDE treatment was the most effective in preserving carbohydrates. Shorter exposure (90 s) minimized early losses in the peel, while longer exposure (120–150 s) significantly enhanced sugar retention in both the peel and juice, suggesting plasma‐modulated metabolic activity.

These findings align with previous studies, which show that cold storage induces early carbohydrate loss due to respiration, followed by metabolic shifts that promote sugar accumulation (Niu et al. 2024; Yu et al. 2022). Plasma treatment has been reported to elevate ATP levels and enhance sugar metabolism (Jiang et al. 2007; Li, Li, Ji, et al. 2019), and it also modifies pectin structure and cell wall integrity, facilitating sugar solubilization (Momeni et al. 2018). Additionally, carbohydrate fluctuations may reflect the reallocation of sugar resources toward phenolic biosynthesis, which contributes to fruit defense mechanisms and improved postharvest stability (Jacobo‐Velázquez et al. 2011). Overall, the observed fluctuations in total carbohydrate content following plasma treatment are likely the result of both direct metabolic alterations and secondary biochemical responses—particularly phenolic biosynthesis—which enhance fruit defense mechanisms and contribute to improved postharvest stability.

### 3.9 Tannin Content in Lime Juice

**FIGURE 10:**
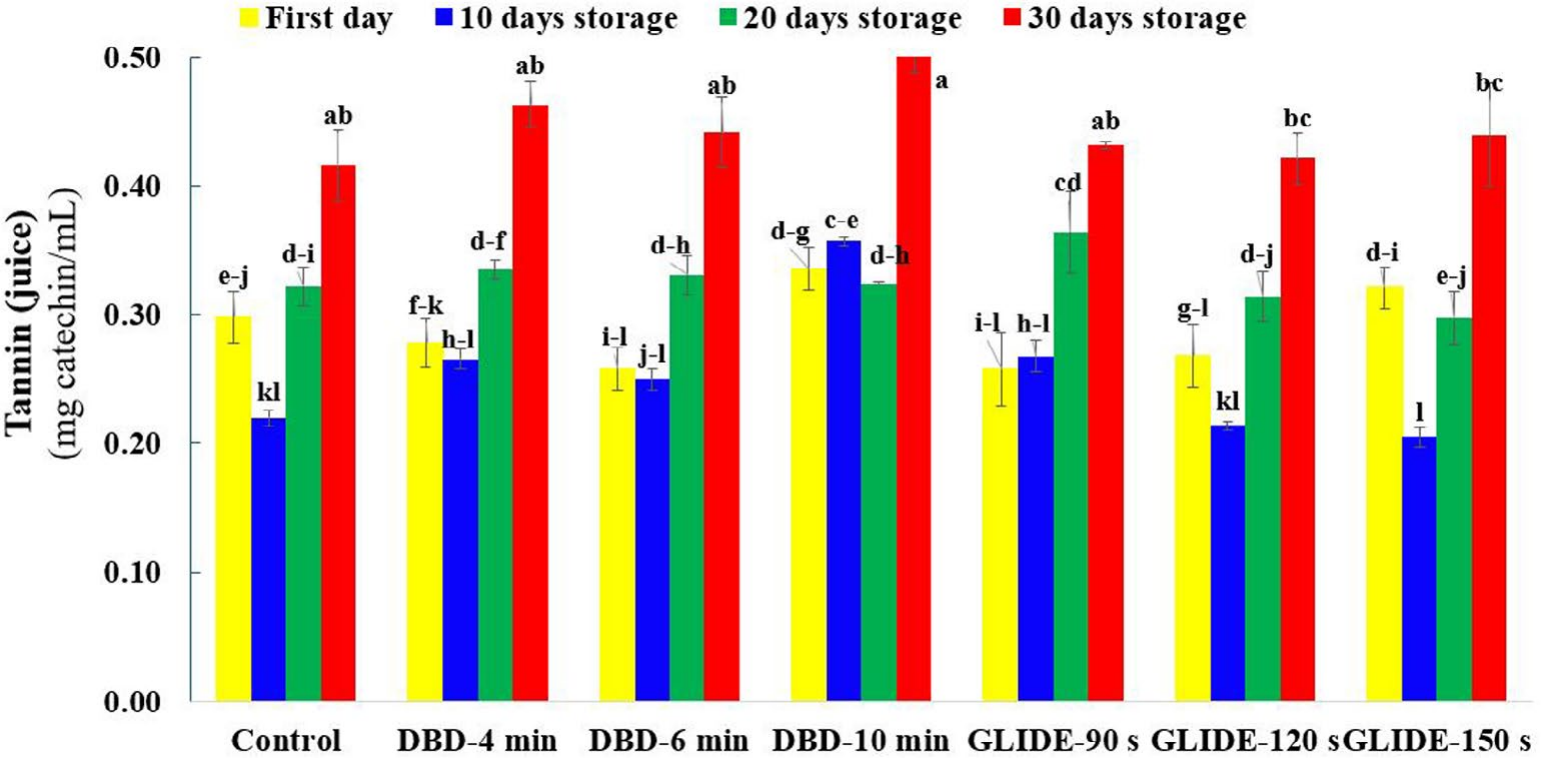
Total tannin content of lime fruit juice, expressed as catechin equivalents, in control and plasma‐treated samples during the storage period.

The analysis of tannin content in lime juice during storage following CP pretreatment using DBD and gliding arc plasma reveals significant variations influenced by both plasma type and storage duration. Tannins, high‐molecular‐weight phenolics responsible for the astringency and cloudiness of fruit juice, can be categorized into hydrolyzable and condensed tannins. In this study, condensed tannins, which are composed of flavonoid polymers and exhibit higher resistance to degradation, were measured. The results demonstrate that tannin levels, although initially low across all samples, increased progressively throughout storage, with the highest concentrations observed after 30 days (Figure 10).

DBD‐treated samples, particularly those exposed for 10 min, show the most substantial rise in tannin content, approximately 21% higher than the control. This increase is likely attributed to the plant's stress response to plasma‐induced reactive oxygen species (ROS), which can trigger secondary metabolite synthesis, including tannins. The GLIDE‐treated samples also showed a similar trend, though the increase in tannin content was slightly lower than that observed in DBD‐treated samples. This discrepancy may stem from differences in plasma characteristics, as DBD treatment, due to its prolonged exposure time, may have induced more extensive structural modifications leading to greater tannin formation.

These findings contrast with those of Herceg et al. (2016), who reported a reduction in tannin content following plasma treatment in pomegranate juice, attributed to plasma‐induced hydrolysis and depolymerization of ellagitannins into ellagic acid. The apparent contradiction may be explained by the distinct nature of tannins analyzed in each study. Unlike hydrolyzable tannins, condensed tannins are more stable and resistant to degradation, suggesting that plasma treatment, rather than breaking them down, may have stimulated their biosynthesis as part of the fruit's stress adaptation mechanism. Therefore, the observed increase in tannin content following plasma exposure is likely a combined effect of plasma‐induced biochemical modifications and prolonged storage, both of which influence the phenolic metabolism of lime juice.

## 4 Conclusion

This study is the first to compare two distinct CP treatment strategies—prolonged low‐intensity DBD and short high‐intensity GLIDE—and highlight their differential effects on the antioxidant activity of lime fruit. The results demonstrate that both DBD and GLIDE plasma treatments significantly improved the antioxidant properties of lime fruit by modifying surface chemistry and stimulating metabolic responses. After 30 days of storage, both treatments notably enhanced antioxidant capacity. Specifically, DBD‐10 min increased juice total flavonoids by 69.70% and peel total flavonoids by 83.66%, while GLIDE‐150 s increased juice total flavonoids by 44.16% and peel total flavonoids by 93.32%. Both treatments also improved peel antioxidant capacity by approximately 11%, compared to the control. Among the treatments, DBD‐10 min was the most effective at enhancing bioactive compounds, including vitamin C, total phenolics, flavonoids, and tannins, particularly in the peel. This effect is attributed to the prolonged exposure, which induces deeper biochemical modifications. In contrast, GLIDE plasma, applied for shorter durations, also increased bioactive content, though to a lesser extent, while it better preserved carbohydrate content. Future research should focus on optimizing plasma parameters to balance the enhancement of nutritional properties with the preservation of fruit quality for commercial applications. Although challenges related to scalability, high capital costs, and regulatory approval remain, the long‐term benefits of CP technology—such as reduced food waste, enhanced nutritional value, and the potential to produce functional “superfood” fruits—could justify the investment.

## Author Contributions

**Ghazal Razazian:** data curation (equal), formal analysis (equal), investigation (equal), methodology (equal). **Mohsen Dalvi‐Isfahan:** supervision (equal), project administration (equal), conceptualization (equal), writing – review and editing (equal), validation (equal). **Saeideh Mohtashami:** data curation (equal), formal analysis (equal), methodology (equal), supervision (equal), validation (equal), visualization (equal).

## Ethics Statement

The authors have nothing to report.

## Conflicts of Interest

The authors declare no conflicts of interest.

## Data Availability

The data that support the findings of this study are available on reasonable request from the corresponding author.

## References

[fsn371091-bib-0001] Abidi, N. , L. Cabrales , and C. H. Haigler . 2014. “Changes in the Cell Wall and Cellulose Content of Developing Cotton Fibers Investigated by FTIR Spectroscopy.” Carbohydrate Polymers 100: 9–16. 10.1016/j.carbpol.2013.01.074. 24188832

[fsn371091-bib-0002] Ahmadnia, M. , M. Sadeghi , R. Abbaszadeh , and H. R. Ghomi Marzdashti . 2021. “Decontamination of Whole Strawberry via Dielectric Barrier Discharge Cold Plasma and Effects on Quality Attributes.” Journal of Food Processing & Preservation 45, no. 1: e15019.

[fsn371091-bib-0003] Akaber, S. , Y. Ramezan , and M. Reza Khani . 2024. “Effect of Post‐Harvest Cold Plasma Treatment on Physicochemical Properties and Inactivation of Penicillium Digitatum in Persian Lime Fruit.” Food Chemistry 437: 137616. 37866339 10.1016/j.foodchem.2023.137616

[fsn371091-bib-0004] Alashti, F. J. , F. Sohbatzadeh , S. Ahmadian , R. E. Kenari , and E. Nazifi . 2024. “Impact of Atmospheric Cold Plasma Pretreatment on Morphology, Structure, and Chemical Properties of Clove ( *Syzygium aromaticum* ).” LWT—Food Science and Technology 191: 115639. 10.1016/j.lwt.2023.115639.

[fsn371091-bib-0005] Bakhshandeh, E. , M. Zeraatpisheh , A. Soleimani , and R. Francaviglia . 2022. “Land Use Conversion, Climate Change and Soil Organic Carbon: Modeling a Citrus Garden Chronosequence in Northern Iran.” Geoderma Regional 30: e00559.

[fsn371091-bib-0006] Birania, S. , A. K. Attkan , S. Kumar , N. Kumar , and V. K. Singh . 2022. “Cold Plasma in Food Processing and Preservation: A Review.” Journal of Food Process Engineering 45, no. 9: e14110.

[fsn371091-bib-0007] Broadhurst, R. B. , and W. T. Jones . 1978. “Analysis of Condensed Tannins Using Acidified Vanillin.” Journal of the Science of Food and Agriculture 29, no. 9: 788–794.

[fsn371091-bib-0008] Bursać Kovačević, D. , P. Putnik , V. Dragović‐Uzelac , S. Pedisić , A. Režek Jambrak , and Z. Herceg . 2016. “Effects of Cold Atmospheric Gas Phase Plasma on Anthocyanins and Color in Pomegranate Juice.” Food Chemistry 190: 317–323. 26212976 10.1016/j.foodchem.2015.05.099

[fsn371091-bib-0009] Chen, Y.‐Q. , C. Jun‐Hu , and D.‐W. Sun . 2020. “Chemical, Physical and Physiological Quality Attributes of Fruit and Vegetables Induced by Cold Plasma Treatment: Mechanisms and Application Advances.” Critical Reviews in Food Science and Nutrition 60, no. 16: 2676–2690. 32876477 10.1080/10408398.2019.1654429

[fsn371091-bib-0010] Dalvi‐Isfahan, M. , and M. Mahmoodi‐Eshkaftaki . 2024. “Potential Applications of Atmospheric‐Pressure Dielectric Barrier Discharge Cold Plasma for Fruit Preservation: Advantages, Effects on Quality Characteristics, and Limitations.” Innovative Food Science and Emerging Technologies 94: 103675.

[fsn371091-bib-0011] Dasan, B. G. , and I. H. Boyaci . 2018. “Effect of Cold Atmospheric Plasma on Inactivation of Escherichia Coli and Physicochemical Properties of Apple, Orange, Tomato Juices, and Sour Cherry Nectar.” Food and Bioprocess Technology 11, no. 2: 334–343.

[fsn371091-bib-0012] Denoya, G. I. , G. Pataro , and G. Ferrari . 2020. “Effects of Postharvest Pulsed Light Treatments on the Quality and Antioxidant Properties of Persimmons During Storage.” Postharvest Biology and Technology 160: 111055.

[fsn371091-bib-0013] Dong, X. Y. , and Y. L. Yang . 2019. “A Novel Approach to Enhance Blueberry Quality During Storage Using Cold Plasma at Atmospheric Air Pressure.” Food and Bioprocess Technology 12, no. 8: 1409–1421. 10.1007/s11947-019-02305-y.

[fsn371091-bib-0014] Fernandes, F. A. N. , and S. Rodrigues . 2021. “Cold Plasma Processing on Fruits and Fruit Juices: A Review on the Effects of Plasma on Nutritional Quality.” PRO 9, no. 12: 2098.

[fsn371091-bib-0015] Ghomi, H. , N. N. Safa , and M. R. Golghand . 2017. “Generation of Dielectric Barrier Discharge Plasma Using a Modulated Voltage.” United States Patent Application Publication 15: 409–457.

[fsn371091-bib-0016] González‐Estrada, R. R. , E. Carvajal‐Millán , J. A. Ragazzo‐Sánchez , P. U. Bautista‐Rosales , and M. Calderón‐Santoyo . 2017. “Control of Blue Mold Decay on Persian Lime: Application of Covalently Cross‐Linked Arabinoxylans Bioactive Coatings With Antagonistic Yeast Entrapped.” LWT‐ Food Science and Technology 85: 187–196.

[fsn371091-bib-0017] Grzegorzewski, F. , S. Rohn , L. W. Kroh , M. Geyer , and O. Schlüter . 2010. “Surface Morphology and Chemical Composition of Lamb's Lettuce ( *Valerianella locusta* ) After Exposure to a Low‐Pressure Oxygen Plasma.” Food Chemistry 122, no. 4: 1145–1152.

[fsn371091-bib-0018] He, X. , T. Sun , W. Zhang , et al. 2025. “Cold Plasma Treatment Maintains Antioxidant Capacity and Cell Membrane Integrity in Apricot Fruit by Inducing Reactive Oxygen Species Scavenging Systems.” Postharvest Biology and Technology 230: 113815. 10.1016/j.postharvbio.2025.113815.

[fsn371091-bib-0019] Herceg, Z. , D. B. Kovačević , J. G. Kljusurić , A. R. Jambrak , Z. Zorić , and V. Dragović‐Uzelac . 2016. “Gas Phase Plasma Impact on Phenolic Compounds in Pomegranate Juice.” Food Chemistry 190: 665–672. 26213024 10.1016/j.foodchem.2015.05.135

[fsn371091-bib-0020] Huang, C.‐C. , J. S.‐B. Wu , J.‐S. Wu , and Y. Ting . 2019. “Effect of Novel Atmospheric‐Pressure Jet Pretreatment on the Drying Kinetics and Quality of White Grapes.” Journal of the Science of Food and Agriculture 99, no. 11: 5102–5111. 10.1002/jsfa.9754. 30982968

[fsn371091-bib-0021] Huynh, D. T. , M. T. N. Vo , and T. C. Kha . 2023. “Enriching the Bioactive Components and Antioxidant Capacity of Concentrated Lime Juices Prepared by Cryogenic and Vacuum Processes.” PRO 11, no. 7: 1883.

[fsn371091-bib-0022] Jacobo‐Velázquez, D. A. , G. B. Martínez‐Hernández , S. del C. Rodríguez , C.‐M. Cao , and L. Cisneros‐Zevallos . 2011. “Plants as Biofactories: Physiological Role of Reactive Oxygen Species on the Accumulation of Phenolic Antioxidants in Carrot Tissue Under Wounding and Hyperoxia Stress.” Journal of Agricultural and Food Chemistry 59, no. 12: 6583–6593. 21553806 10.1021/jf2006529

[fsn371091-bib-0023] Jiang, Y. , Y. Jiang , H. Qu , X. Duan , Y. Luo , and W. Jiang . 2007. “Energy Aspects in Ripening and Senescence of Harvested Horticultural Crops.” Stewart Postharvest Review 3: 1–5.

[fsn371091-bib-0024] Kurniawan, Y. S. , M. R. G. Fahmi , and L. Yuliati . 2020. “Isolation and Optical Properties of Natural Pigments From Purple Mangosteen Peels.” IOP Conference Series: Materials Science and Engineering 833, no. 1: e012018. 10.1088/1757-899X/833/1/012018.

[fsn371091-bib-0025] Li, M. , X. Li , C. Han , N. Ji , P. Jin , and Y. Zheng . 2019. “Physiological and Metabolomic Analysis of Cold Plasma Treated Fresh‐Cut Strawberries.” Journal of Agricultural and Food Chemistry 67, no. 14: 4043–4053. 30883111 10.1021/acs.jafc.9b00656

[fsn371091-bib-0026] Li, M. , S. Zhao , Z. Kong , et al. 2023. “Preservation of Citrus Fruit, and Dissipation by Diffusion and Degradation of Postharvest Pesticides During Storage.” Journal of Food Composition and Analysis 122: 105456.

[fsn371091-bib-0027] Li, X. , M. Li , N. Ji , et al. 2019. “Cold Plasma Treatment Induces Phenolic Accumulation and Enhances Antioxidant Activity in Fresh‐Cut Pitaya (*Hylocereus undatus*) Fruit.” LWT ‐ Food Science and Technology 115: 108447.

[fsn371091-bib-0028] Lin, Y. S. , W. Y. Huang , P. Y. Ho , et al. 2020. “Effects of Storage Time and Temperature on Antioxidants in Juice From *Momordica charantia* L. and *Momordica charantia* L. Var. Abbreviata Ser.” Molecules 25, no. 16: 3614. 32784816 10.3390/molecules25163614 PMC7464073

[fsn371091-bib-0029] Menichini, F. , R. Tundis , M. Bonesi , et al. 2009. “The Influence of Fruit Ripening on the Phytochemical Content and Biological Activity of *Capsicum chinense* Jacq. cv Habanero.” Food Chemistry 114, no. 2: 553–560.

[fsn371091-bib-0030] Misra, N. N. , S. K. Pankaj , J. M. Frias , K. M. Keener , and P. J. Cullen . 2015. “The Effects of Nonthermal Plasma on Chemical Quality of Strawberries.” Postharvest Biology and Technology 110: 197–202.

[fsn371091-bib-0031] Mohammadi, M. , S. Rastegar , and A. Rohani . 2024. “Enhancing Mexican Lime ( *Citrus aurantifolia* cv.) Shelf Life With Innovative Edible Coatings: Xanthan Gum Edible Coating Enriched With Spirulina Platensis and Pomegranate Seed Oils.” BMC Plant Biology 24, no. 1: 906. 39350034 10.1186/s12870-024-05606-3 PMC11440758

[fsn371091-bib-0032] Momeni, M. , M. Tabibiazar , S. Khorram , et al. 2018. “Pectin Modification Assisted by Nitrogen Glow Discharge Plasma.” International Journal of Biological Macromolecules 120: 2572–2578. 30195607 10.1016/j.ijbiomac.2018.09.033

[fsn371091-bib-0033] Najwa, R. , and A. Azlan . 2017. “Comparison of Vitamin C Content in Citrus Fruits by Titration and High Performance Liquid Chromatography (HPLC) Methods.” International Food Research Journal 24: 726–733.

[fsn371091-bib-0034] Navaratne, S. , and C. Sandaruwani . 2014. “Preservation of Lime Fruits Under Modified Atmospheric Condition Created in a Sand Bed.” International Journal of Innovative Research in Technology 1: 85–91.

[fsn371091-bib-0035] Niu, X.‐X. , L.‐Z. Deng , H. Wang , et al. 2024. “Transformation of Cell Wall Pectin Profile During Postharvest Ripening Process Alters Drying Behavior and Regulates the Sugar Content of Dried Plums.” Food Chemistry 458: 140093. 38943960 10.1016/j.foodchem.2024.140093

[fsn371091-bib-0036] Oke, F. , B. Aslim , S. Ozturk , and S. Altundag . 2009. “Essential Oil Composition, Antimicrobial and Antioxidant Activities of Satureja Cuneifolia Ten.” Food Chemistry 112, no. 4: 874–879.

[fsn371091-bib-0037] Popova, M. , V. Bankova , D. Butovska , et al. 2004. “Validated Methods for the Quantification of Biologically Active Constituents of Poplar‐Type Propolis.” Phytochemical Analysis 15, no. 4: 235–240. 15311843 10.1002/pca.777

[fsn371091-bib-0038] Puligundla, P. , T. Lee , and C. Mok . 2018. “Effect of Intermittent Corona Discharge Plasma Treatment for Improving Microbial Quality and Shelf Life of Kumquat (*Citrus japonica*) Fruits.” LWT–Food Science and Technology 91: 8–13.

[fsn371091-bib-0039] Rapisarda, P. , M. L. Bianco , P. Pannuzzo , and N. Timpanaro . 2008. “Effect of Cold Storage on Vitamin C, Phenolics and Antioxidant Activity of Five Orange Genotypes [ *Citrus sinensis* (L.) Osbeck].” Postharvest Biology and Technology 49, no. 3: 348–354.

[fsn371091-bib-0040] Sarangapani, C. , G. O'Toole , P. J. Cullen , and P. Bourke . 2017. “Atmospheric Cold Plasma Dissipation Efficiency of Agrochemicals on Blueberries.” Innovative Food Science and Emerging Technologies 44: 235–241.

[fsn371091-bib-0041] Sreelakshmi, V. P. , S. E. Vendan , and P. S. Negi . 2024. “The Effect of Cold Plasma Treatment on Quality Attributes and Shelf Life of Apples.” Postharvest Biology and Technology 214: 112975.

[fsn371091-bib-0042] Tappi, S. , G. Gozzi , L. Vannini , et al. 2016. “Cold Plasma Treatment for Fresh‐Cut Melon Stabilization.” Innovative Food Science and Emerging Technologies 33: 225–233.

[fsn371091-bib-0043] Wojdyło, A. , J. Oszmiański , and R. Czemerys . 2007. “Antioxidant Activity and Phenolic Compounds in 32 Selected Herbs.” Food Chemistry 105, no. 3: 940–949.

[fsn371091-bib-0044] Won, M. Y. , S. J. Lee , and S. C. Min . 2017. “Mandarin Preservation by Microwave‐Powered Cold Plasma Treatment.” Innovative Food Science and Emerging Technologies 39: 25–32.

[fsn371091-bib-0045] Yawut, N. , T. Mekwilai , N. Vichiansan , S. Braspaiboon , K. Leksakul , and D. Boonyawan . 2024. “Cold Plasma Technology: Transforming Food Processing for Safety and Sustainability.” Journal of Agriculture and Food Research 18: 101383.

[fsn371091-bib-0046] Yemm, E. W. , and A. J. Willis . 1954. “The Estimation of Carbohydrates in Plant Extracts by Anthrone.” Biochemical Journal 57, no. 3: 508–514. 13181867 10.1042/bj0570508 PMC1269789

[fsn371091-bib-0047] Yu, J. , Y. Tseng , K. Pham , M. Liu , and D. M. Beckles . 2022. “Starch and Sugars as Determinants of Postharvest Shelf Life and Quality: Some New and Surprising Roles.” Current Opinion in Biotechnology 78: 102844. 36410153 10.1016/j.copbio.2022.102844

[fsn371091-bib-0048] Zargarchi, S. , J. Hornbacher , S. M. Afifi , et al. 2024. “Exploring the Impact of Cold Plasma Treatment on the Antioxidant Capacity, Ascorbic Acid, Phenolic Profile, and Bioaccessibility of Fruits and Fruit Juices.” Food Frontiers 5, no. 3: 1108–1125.

[fsn371091-bib-0049] Zhou, B. , H. Zhao , X. Yang , and J.‐H. Cheng . 2024. “Versatile Dielectric Barrier Discharge Cold Plasma for Safety and Quality Control in Fruits and Vegetables Products: Principles, Configurations and Applications.” Food Research International 196: 115117. 39614520 10.1016/j.foodres.2024.115117

